# Risk Factors of Middle Lobe Torsion in Patients Who Underwent Thoracoscopic Right Upper Lobectomy

**DOI:** 10.5761/atcs.oa.25-00022

**Published:** 2025-04-23

**Authors:** Hidenori Goto, Kozo Nakanishi

**Affiliations:** Department of General Thoracic Surgery, National Hospital Organization, Saitama Hospital, Wako, Saitama, Japan

**Keywords:** right upper lobectomy, middle lobe torsion, risk factors, thoracoscopic surgery

## Abstract

**Purpose:** Lung torsion is a rare postoperative complication of pulmonary resection caused by lobe displacement. This condition leads to bronchial or pulmonary vascular kinking, which results in airway obstruction or blood flow impairment. In particular, middle lobe torsion is commonly observed after right upper lobectomy. However, the conditions under which it occurs remain unclear. This study aimed to identify the risk factors for middle lobe torsion following right upper lobectomy.

**Methods:** From November 2012 to December 2024, 127 patients underwent thoracoscopic right upper lobectomy at our institution. Four patients diagnosed with postoperative middle lobe torsion were classified into the torsion group. These patients were retrospectively compared with those without torsion.

**Results:** Simultaneous partial middle lobe resection and the number of staples used for interlobar fissure formation between the upper and middle lobes were significantly associated with lung torsion. The cutoff value for the number of staples used in the upper-middle fissure formation was 4, demonstrating fair accuracy.

**Conclusions:** The risk factors for middle lobe torsion after thoracoscopic right upper lobectomy were simultaneous partial resection of the middle lobe and the number of staples used for interlobar fissure formation between the upper and middle lobes.

## Introduction

Lung torsion is a rare postoperative complication of pulmonary resection caused by the rotation of the bronchi or pulmonary vessels due to displacement, leading to airway obstruction and vascular impairment.^1)^ The incidence rate of lung torsion after lobectomy ranges from 0.3% to 0.4%, with 74.4% of cases occurring after right upper lobectomy, among which middle lobe torsion is the most common.^2,3)^ The etiological factors contributing to this complication remain unclear; however, its mortality rate is 8.3%. This finding emphasizes the potentially fatal severity of the condition and underscores the importance of preventive measures following pulmonary resection.^2)^ However, considering its rarity, risk factors should be identified to narrow down the cases where preventive strategies should be considered. Thus, this study aimed to investigate the risk factors of middle lobe torsion, which is the most frequent complication following right upper lobectomy.

## Materials and Methods

### Methods

A database comprising consecutive patients who underwent thoracoscopic lobectomy at our department between November 2012 and December 2024 was retrospectively reviewed. The diagnosis of postoperative lobe torsion was determined through imaging examinations. The primary diagnostic methods included chest radiography, chest computed tomography (CT), and bronchoscopy, which confirmed bronchial or vascular narrowing or obstruction, along with associated consolidation.^2)^ During this period, 441 patients underwent thoracoscopic lobectomy, of whom 4 (0.9%) were diagnosed with postoperative lobar torsion. All 4 patients presented with middle lobe torsion after right upper lobectomy. These patients were classified into the torsion group. The remaining 123 patients who underwent right upper lobectomy during the same period were included in the non-torsion group. The following clinical characteristics and intraoperative factors were compared between the 2 groups. The clinical characteristics included age at the time of surgery, sex, pathological diagnosis, smoking history, and forced expiratory volume in 1 second percent (FEV1%). The intraoperative factors included the surgical approach (uniport or multiport), surgical duration, blood loss volume, presence of bronchoplasty, simultaneous partial resection of other lobes, extent of nodal dissection, frequency of complete oblique fissures between the middle and lower lobes processed during surgery, and the number of staples (staple lengths: 30/45 mm) used for interlobar fissure formation between the upper and middle lobes and between the upper and lower lobes. Notably, preventive measures such as pulmonary ligament division to prevent middle lobe torsion were not performed during right upper lobectomy at our department.

### Statistical analysis

Continuous variables were expressed as the mean ± standard deviation. The Wilcoxon rank-sum test was used to compare continuous data. Pearson’s chi-square test was applied to examine categorical data. Multivariate logistic regression analysis was performed to identify the risk factors. To decrease the risk of overlooking significant factors, variables with a *p* value of <0.10 in the univariate analysis were included in the multivariate analysis. Adjusted odds ratios (ORs), 95% confidence intervals, and *p* values for significant risk factors were reported. Receiver-operating characteristic curve analysis was performed to determine the cutoff values for quantitative data variables. A *p* value of <0.05 was used to indicate statistically significant differences. The EZR software was used to perform statistical analyses.^4)^

## Results

**Fig. 1:**
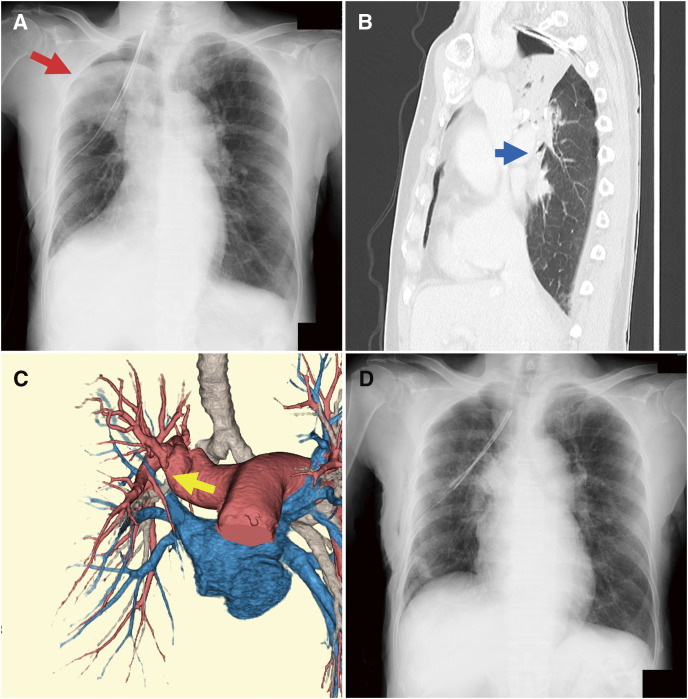
(**A**) Postoperative chest radiograph showing an infiltration shadow (red arrow) in the right upper lung field. (**B**) Postoperative sagittal CT scan showing interruption of the middle lobe bronchus (blue arrow) with atelectasis. (**C**) Three-dimensional CT scan showing blurring of the middle lobe bronchus (yellow arrow). (**D**) A chest radiograph on postoperative day 1 showing good extension of the lungs. CT: computed tomography

**Table 1** shows the postoperative course of the 4 patients in the torsion group. None of the patients in the torsion group presented with middle lobe torsion on the intraoperative videos just before the end of surgery or showed abnormal findings such as consolidation on chest radiography taken immediately after surgery. The diagnosis of middle lobe torsion was based on chest radiography and CT scans performed on or after postoperative day 1 (**Figs. 1A**–**1C**). Detorsion of the middle lobe was performed on the day of diagnosis (**Fig. 1D**). In the detorsion procedure, the ports used in the previous thoracoscopic right upper lobectomy were reused for surgical access. No special interventions, such as anticoagulant therapy, were implemented before or after the reoperation.

**Table 1:** Postoperative course in the LT group

Case	Age/sex	Pathological diagnosis/operative procedure	Postoperative day until reoperation (days)	Intraoperative findings	Surgical technique	Post-resurgery complications	Post-resurgery hospital stay (days)
				Direction and degree of torsion/condition of the right middle lobe			
1	64/F	Metastatic lung tumor /MVATS RUL + ND1a + PR of the right middle lobe	7	Clockwise, partial/congestion	Reposition	—	6
2	73/M	Primary lung cancer/UVATS RUL + ND2a-1	1	Counterclockwise, complete/congestion	Repositioning with fixation by suturing the middle and lower lobes together	—	7
3	82/F	Primary lung cancer/UVATS RUL + ND1a	1	Counterclockwise, complete/congestion	Repositioning with fixation by suturing the middle and lower lobes together	—	11
4	71/F	Primary lung cancer/UVATS RUL + ND2a-1	1	Clockwise, complete/congestion	Repositioning with fixation by suturing the middle and lower lobes together	Resurgery for pulmonary fistula after reoperation	34

PR: partial resection; ND: node dissection; MVATS: multiportal video-assisted thoracoscopic surgery; UVATS: uniportal video-assisted thoracoscopic surgery; RUL: right upper lobectomy; LT: lung torsion

**Table 2** presents the characteristics of the patients in the torsion and non-torsion groups. The 2 groups did not significantly differ in terms of clinical characteristics such as age, sex, pathological diagnosis, smoking history, and FEV1%. Similarly, there were no significant differences in intraoperative factors, including surgical approach, surgical duration, blood loss volume, extent of node dissection (ND), frequency of bronchoplasty, simultaneous partial lower lobe resection, and complete oblique fissures in the middle and lower lobes. In addition, the number of staples used for fissure formation between the upper and lower lobes did not significantly differ between the 2 groups. However, the torsion group had a higher frequency of simultaneous partial middle lobe resection (*p* = 0.092) and a greater number of staples used for fissure formation between the upper and middle lobes (*p* = 0.068) compared to the non-torsion group. Moreover, there were no abnormalities in the branching or course of the right pulmonary vessels or bronchi in the torsion group. Based on the multivariate logistic regression analysis of intraoperative factors, simultaneous partial middle lobe resection (*p* = 0.007) and the number of staples used for fissure formation between the upper and middle lobes (*p* = 0.014) were significantly associated with middle lobe torsion (**Table 3**).

**Table 2:** Characteristics of the LT and NLT groups

	LT group (n = 4)	NLT group (n = 123)	*p* value
Clinical characteristics of the patients			
Age (years)	72.5 ± 7.4	70.1 ± 8.6	0.663
Sex			0.307
Male	1 (25.0)	73 (59.3)	
Female	3 (75.0)	50 (40.7)	
Smoking history (pack-years)	16.3 ± 26.3	28.7 ± 31.3	0.502
FEV1%	73.0 ± 11.9	75.2 ± 10.1	0.592
Pathological diagnosis			0.122
Primary lung cancer	3 (75.0)	120 (97.6)	
Metastatic lung tumor	1 (25.0)	2 (1.6)	
Benign tumor	0 (0.0)	1 (0.8)	
Intraoperative factors			
Approach			0.298
Multiple VATS	1 (25.0)	77 (62.6)	
Uniportal VATS	3 (75.0)	46 (37.4)	
Extent of ND			0.224
I	2 (50.0)	27 (22.0)	
II	2 (50.0)	96 (78.0)	
Simultaneous PR of the other lobes			
Middle lung lobe	1 (33.3)	3 (2.4)	0.092
Lower lung lobe	0 (0.0)	4 (3.3)	1.000
Bronchoplasty	0 (0.0)	1 (0.8)	1.000
Surgical duration (min)	287 ± 48	329 ± 72	0.197
Blood loss volume (mL)	156 ± 103	115 ± 126	0.297
Number of staples used (n)			
Upper and middle lobe fissures	3.75 ± 2.06	2.11 ± 1.47	0.068
Upper and lower lobe fissures	1.25 ± 1.50	1.02 ± 1.17	0.752
Complete oblique fissure	4 (100.0)	70 (56.9)	0.140

FEV1%: forced expiratory volume in 1 second percent.

Values were expressed as the numbers (percentages) or means ± standard deviation. The *p* values were obtained using the Wilcoxon rank-sum test. The other *p* values were achieved using the Pearson’s chi-square test.

PR: partial resection; ND: node dissection; VATS: video-assisted thoracoscopic surgery; LT: lung torsion; NLT: non-lung torsion

**Table 3:** Multiple logistic regression analysis of simultaneous PR of the middle lung lobe and the number of staples used for the upper and middle lobe fissures

Variables	OR	95% CI	*p* value
Simultaneous PR of the middle lung lobe	204.0	4.30–970	0.007
Number of staples used for the upper and middle lobe fissures	3.22	1.27–8.17	0.014

CI: confidence interval; OR: odds ratio; PR: partial resection

**Fig. 2:**
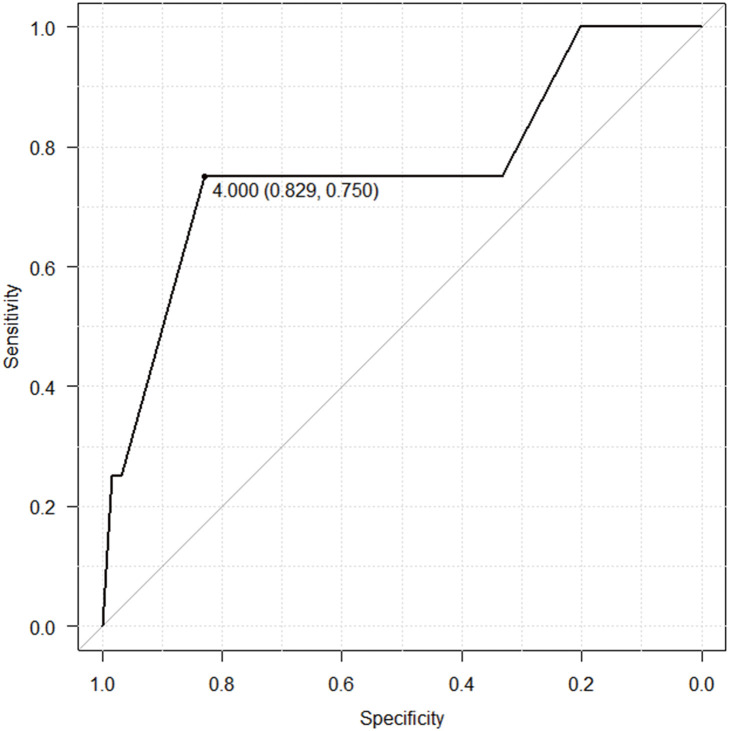
The receiver-operating characteristic curve for the number of staples used for the upper and middle lobe fissure. In the analysis of the number of staples used, 4 staples were determined as the optimal cutoff value (area under the curve: 0.764)

The receiver-operating characteristic curve analysis (**Fig. 2**) revealed that the cutoff value for the number of staples used for fissure formation between the upper and middle lobes was 4, demonstrating fair accuracy.

## Discussion

Lung torsion is an extremely rare but life-threatening complication following pulmonary resection.^1,2)^ Factors such as complete interlobar fissures, elongated pulmonary pedicles, pneumothorax, atelectasis, pleural effusion,^5)^ and division of the pulmonary ligament^6)^ are potential contributors to the development of lung torsion. However, its precise etiology remains unclear.Minimally invasive approaches, such as video-assisted thoracoscopic surgery and robot-assisted thoracoscopic surgery,^7)^ may reduce postoperative adhesion formation and thereby increase the risk of lung torsion as a possible complication after pulmonary resection. Nonetheless, this association has not been definitively established.^2)^

Our study showed that simultaneous partial resection of the middle lobe and the number of staples used for interlobar fissure formation between the upper and middle lobes are significant risk factors for middle lobe torsion after video-assisted thoracoscopic surgery right upper lobectomy. The use of staples on the middle lobe may induce deformation, thereby predisposing the middle lobe to rotation and subsequent torsion due to displacement during postoperative lung expansion. Based on these findings, the degree of middle lobe deformation plays an important role in the development of torsion.

The use of staples on the lung parenchyma can lead to deformation, with the degree of deformation increasing as the thickness of the parenchyma being stapled increases. When forming interlobar fissures, more severe incomplete fissures require stapling of the thicker parenchyma over longer distances, resulting in an increased number of staples used. Furthermore, in partial resection of the middle lobe, achieving sufficient margins for lesion resection often results in significant deformation of the remaining middle lobe. Compared with interlobar fissure formation for anatomical resections, partial resection of the middle lobe is likely to induce greater deformation, as suggested by the OR in our analysis. This result is consistent with previous reports indicating that partial resection of the middle lobe is a potential risk factor for postoperative middle lobe torsion.^8)^ To the best of our knowledge, this study is the first to report that the number of staples used for upper-middle lobe fissure formation is a potential contributor to postoperative middle lobe torsion.

A complete oblique fissure between the middle and lower lobes, a structural prerequisite for torsion, was observed in all 4 cases in the torsion group; however, no significant difference was found compared to the non-torsion group (**Table 2**). Although the presence of a complete oblique fissure alone did not lead to postoperative middle lobe torsion, its role as a necessary condition for its development cannot be ruled out. Therefore, it would be reasonable to consider preventive measures for middle lobe torsion in cases where a complete oblique fissure between the middle and lower lobes is present, particularly when a high number of staples are used for upper-middle lobe fissure formation due to simultaneous partial middle lobectomy or incomplete fissure between the upper and middle lobes.

The treatment for lobe torsion includes either detorsion or resection of the torsed lobe. However, the criteria for treatment selection remain undefined.^2)^ Detorsion of infarcted lung tissue may lead to secondary embolism^9,10)^ caused by the release of emboli from obstructed pulmonary veins, acute inflammatory responses triggered by inflammatory mediators from twisted segments,^11)^ acute respiratory distress syndrome, and potentially multiorgan dysfunction.^12)^ Ideally, detorsion should be performed to salvage ischemic lung tissue before the development of irreversible embolism.^13)^ However, in cases of irreversible embolism, resection of the torsed lobe is required. The lack of objective criteria for assessing torsed lungs makes this evaluation dependent on the surgeon’s interpretation. The use of intraoperative imaging with indocyanine green fluorescence has been reported. However, due to its low sensitivity, its use as a supplementary diagnostic tool is limited.^14)^

The time to reoperation is considered an important factor in the treatment selection for lung torsion; however, the optimal timing for detorsion remains unclear. Imaging findings, as the primary diagnostic method, may lead to misdiagnosis as pulmonary hematoma, pneumonia, or atelectasis, potentially delaying appropriate treatment. A report summarizing the treatment outcomes of 103 patients with postoperative lung torsion found that the median interval from the initial surgery to torsion was 3 days.^2)^ Among these cases, 69 patients (67.0%) underwent resection of the torsed lung, whereas 34 patients (33.0%) were treated with detorsion. However, there was no significant difference in mortality between the 2 interventions. Since this report was retrospective and infarcted lesions were often treated with lung resection, it may not sufficiently address this issue. Nevertheless, considering that lung resection for torsed lungs may offer a favorable survival rate, it is essential to resist the temptation to perform detorsion in cases where hemorrhagic infarction is observed due to delayed diagnosis.

In our cases, treatment decisions were based on thoracoscopic findings. Three patients underwent reoperation on postoperative day 1 and presented with congestion in the torsed middle lobe, and detorsion was performed. One patient underwent late resurgery on postoperative day 7 due to a diagnostic delay. Partial congestion was observed, and detorsion was performed. In all cases, the color and texture of the torsed lung improved after detorsion, without subsequent multiorgan dysfunction or embolism after resurgery.

Considering the challenges faced in selecting the appropriate treatment for lung torsion, prevention is significantly important. According to a consensus information, the key preventive measures include proper positioning of the residual lobes and reducing their mobility before chest closure.^2)^ Previous studies have proposed various methods for fixation, which include the use of sutures, staples, glue, and pleural flaps.^5,15–17)^ However, the rarity of this complication has hindered the evaluation of these preventive strategies.^18)^ Each method has its advantages and disadvantages. For example, interlobar suturing is cost-effective but may lead to postoperative air leaks or bleeding.^16)^

In our study, for case 4 (**Table 1**), fixation was performed by suturing the middle and lower lobes together. However, a delayed pneumothorax attributed to a postoperative pulmonary fistula caused dehiscence at the staple line. Although recurrent torsion was prevented, surgical repair of the fistula was required. These findings underscore the need for further accumulation of cases to refine preventive strategies based on our study results.

## Conclusion

The risk factors for middle lobe torsion after thoracoscopic right upper lobectomy were simultaneous partial resection of the middle lobe and the number of staples used for interlobar fissure formation between the upper and middle lobes.

## Declarations

### Ethics approval and consent to participate

The current study was conducted in accordance with the Declaration of Helsinki and was approved by the Research Ethics Committee of Saitama Hospital (approval number: R2022-06).

### Funding

No funding was received for this study.

### Data availability

The datasets analyzed during the current study are available from the corresponding author upon reasonable request.

### Authors’ contributions

Conception and design: H Goto; Administrative support: None; Provision of study materials or patients: H Goto, K Nakanishi; Collection and assembly of data: H Goto, K Nakanishi; Data analysis and interpretation: H Goto; Manuscript writing: H Goto, K Nakanishi; Final approval of manuscript: H Goto, K Nakanishi.

### Disclosure statement

The authors declare that they have no conflicts of interest.
